# The Use of Vinegar to Prevent Vomiting after Chloroform Inhalation

**Published:** 1896-04

**Authors:** 


### The Use of Vinegar to Prevent Vomiting after Chloroform Inhalation.

Lewin (*Revue de Chirurgie*, September, 1895, p. 786), states that he has used his method for the prevention of vomiting after chloroform anæsthesia in 174 cases. These embraced all kinds of operations. In 125 cases success was complete. In the remaining 49 cases vomiting occurred, but it was slight in amount and consisted mostly of glairy mucus. It usually occurred toward evening of the day of operation and ceased by nine o'clock the next morning. The infrequency as well as mildness of the vomiting constituted a practical success, because under the usual chloroformization he has seen vomiting last three days.

Some of the patients vomited because the use of the vinegar cloth was not continued a sufficiently long time.

The following is the method of procedure: A piece of linen the size of a napkin is wet with ordinary vinegar, the surplus vinegar is wrung out and the cloth laid over the chloroform inhaler. This latter is then withdrawn from under the cloth without removing it. This is to prevent the patient respiring air which has not been impregnated with the vinegar vapor. The cloth should remain on the face as long as possible, at least three hours. Should the cloth become dry by the evaporation of the vinegar it should be again moistened. Should the patient desire to expectorate, a cloth or handkerchief is to be slipped beneath the cloth wet with the vinegar. The author claims that it is the sudden inspiration of pure air that causes nausea.—*University Med. Magazine.*

